# The prognosis difference between elderly and younger patients with adrenocortical carcinoma

**DOI:** 10.3389/fgene.2022.1029155

**Published:** 2023-01-04

**Authors:** Shengyin He, Xuemei Huang, Pan Zhao, Peng Zhang

**Affiliations:** ^1^ Department of Urology, West China School of Public Health and West China Fourth Hospital, Institute of Urology, Sichuan University, Chengdu, Sichuan, China; ^2^ Affiliated Traditional Chinese Medicine Hospital of Southwest Medical University, Lu Zhou, Sichuan, China; ^3^ The Affiliated Nanchong Central Hospital of North Sichuan Medical College (University), Nanchong, Sichuan, China; ^3^ Department of Urology, Institute of Urology, West China Hospital, Sichuan University, Chengdu, Sichuan, China

**Keywords:** adrenocortical carcinoma, elderly, survival, prognosis, SEER

## Abstract

**Background and aim:** Adrenocortical carcinoma (ACC) is uncommon in the elderly. This study aimed to compare the surgical prognosis and survival between senior and younger patients. We also explored the factors that were independently related to the survival of elderly patients.

**Methods:** We identified ACC patients between 2010 and 2019 in the Surveillance, Epidemiology, and End Results (SEER) database and applied Kaplan-Meier curves to evaluate the overall survival (OS) and cancer-specific survival (CSS) with log-rank tests. We also used Cox regression analysis to estimate the OS and CSS. The Fine and Gray model with the Gray test was used to measure the cumulative incidence function (CIF) of CSS and other mortality causes of patients in a competing-risks setting.

**Results:** Of 876 patients, 44.06% were elderly. A lower proportion of elderly patients underwent surgery, regional lymph node surgery, and chemotherapy than young patients. Elderly patients also had inferior OS and CSS than younger patients. The 1- and 5-year OS of elderly patients who underwent surgery were 68% [95% confidence interval (CI): 62%–74%] and 30% (95% CI: 24%–38%), and the 1- and 5-year CSS were 73% (95% CI: 67%–80%) and 40% (95% CI: 32%–47%). The factors independently related to worsened survival included age ≥60 [Hazard Ratio (HR): 1.47 (1.24–1.75)], metastatic disease [HR: 1.90 (1.49–2.51)], higher grade [HR: 1.94 (1.08–3.46)] and Network for the Study of Adrenal Tumors (ENSAT) stage [HR: 1.99 (1.48–2.66)].

**Conclusion:** Younger ACC patients had better survival than the elderly. Factors independently related to worsened survival in elderly patients included age ≥60, metastatic disease, higher grade, and European ENSAT stage.

## Introduction

The annual incidence of adrenocortical carcinoma (ACC), a rare tumor, is about 2 per million people worldwide (Schteingart et al., 2005; Paton et al., 2006; Zini et al., 2011). ACC is the second most aggressive endocrine malignancy after anaplastic thyroid cancer but accounts for only around 0.2% of all cancer fatalities in the United States each year (Ng and Libertino, 2003). Women are afflicted more often than men (1.5:1), and 40%–60% of patients show signs of excessive adrenocortical hormones (Libé, 2015). Since most patients have large tumors and advanced diseases when diagnosed, the prognosis for ACC is typically grim. At the time of presentation, 50%–70% of patients have extra-adrenal illness (Fassnacht et al., 2018). Moreover, the clinical outcome for different age groups varies significantly (Hara et al., 2004). ACC has a bimodal age distribution, occurring more frequently before 10 and between 40 and 50 (Else et al., 2014). According to the German ACC Registry, the median age of diagnosis is 46, falling in the fifth-sixth decade (Fassnacht and Allolio, 2009). Adults with ACC tend to be very aggressive, although children with ACC typically have better outcomes than adults. However, it is harder to anticipate tumor behavior in older adults (Kong et al., 2019).

At present, little research has focused on older patients, whereas most concerns pediatric ACC patients. Cancer patients are a unique population, especially older ones. Elderly patients are more susceptible to various medical comorbidities, as well as morbidities and fatalities caused by treatment. Furthermore, in recent studies, the 5-year survival rates of adult patients undergoing surgical excision for ACC ranged between 37% and 39% (Grubbs and Lee, 2009; Ayala-Ramirez et al., 2013). The age at presentation, volume, R1 resections, and the development of metastatic disease were linked to an increased risk of recurrence (Gulack et al., 2016). However, using single-center reviews in these studies has significantly limited their scope.

Hence, in the present study, we examined the outcomes of older ACC patients using a big national database and compared them to younger patients to identify any differences between these two cohorts. Additionally, we explored the factors independently related to survival.

## Methods

### Data source

Data Source Cases were retrieved from the Surveillance, Epidemiology, and End Results (SEER) database covering roughly 28% of the United States population. The SEER program has information regarding the epidemiology of cancer patients. Detailed information about SEER program can be found here: www.seer.cancer.gov.

### Study population

Patients with histologically confirmed ACC (International Classification of Diseases codes: C74.0 and C74.9) between 2010 and 2019 were identified using SEER*STAT software (version 8.4.0). Detailed information about SEER*STAT software can be found here: www.seer.cancer.gov. The European Network for the Study of Adrenal Tumors (ENSAT) staging system is consistent with the eighth American Joint Committee on Cancer (AJCC). Variables such as marital status, race, age at diagnosis, sex, tumor size, grade, laterality, radiotherapy, chemotherapy, ENSAT stage, and regional lymph node surgery were retrieved. First, 1149 ACC patients were extracted. Patients under 18 (*n* = 210) and with an unknown race (*n* = 5), radiotherapy (*n* = 20), chemotherapy (*n* = 23), laterality (*n* = 5), survival status (*n* = 5), and survival month (*n* = 5) were excluded. Finally, 876 patients were included in the study cohort.

### Statistical analysis

Descriptive statistics are presented for patient characteristics. Frequencies and proportions describe categorical variables. The overall survival (OS) and cancer-specific survival (CSS) were evaluated by Kaplan–Meier curves with log-rank tests. “Proportional hazards (PH) assumption” was assessed on the Kaplan–Meier plots constructed. We used Cox regression analysis to estimate the OS and CSS. The Fine and Gray model with the Gray test (Austin and Fine, 2017) was used to measure the cumulative incidence function (CIF) of CSS and other mortality causes of patients in a competing-risks setting. Age cutoff values were determined using the X-tile software v3.6.1 (Yale University, New Haven, CT, United States) (Camp et al., 2004). A *p* < 0.05 was considered statistically significant. Statistical analysis was conducted using SPSS version 22.0 (IBM SPSS Statistics, Chicago, IL, United States) or Empower software (http://www.empowerstats.co-m).

## Results

**FIGURE 1:**
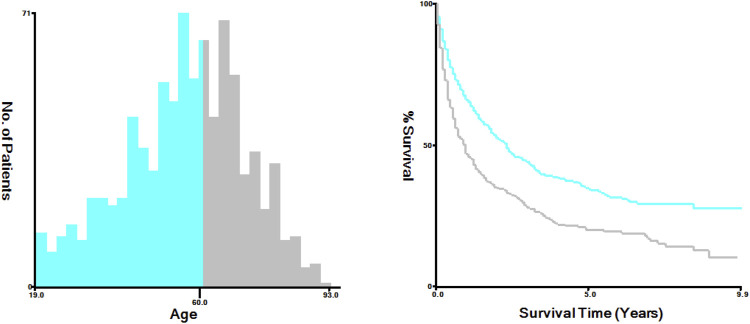
The ACC patients was divided into younger and elderly patients by the X-tile software.

We enrolled 876 patients with newly diagnosed ACC between 2010 and 2019, 538 (61.42%) females and 338 (38.58%) males. According to the X-tile plots (Figure 1), the optimal age cutoff value was 60, which was used to divide the patients into two age groups: <60 [*n* = 490 (55.94%)] and ≥60 [*n* = 386 (44.06%)] (Table 1). Then, we compared the demographic factors and baseline tumor characteristics between the two groups (Table 1). The groups did not differ in the proportion of female patients, ENSAT stage, metastatic disease, radiotherapy, marital status, laterality, tumor size, tumor grade, and race. Compared to younger patients, a lower proportion of elderly patients underwent surgery and received regional lymph node surgery and chemotherapy.

**TABLE 1:** Baseline demographic and tumor characteristics of patients with ACC.

Variables	Age group (years), n (%)		*p*-value
	<60	≥60	
Sex	—	—	0.681
Female	298 (60.82%)	240 (62.18%)	—
Male	192 (39.18%)	146 (37.82%)	—
Follow-up time, median (IQR)	52 (44–59)	58 (47–68)	0.89
Number of patients alive	209 (23.86%)	99 (10.27%)	<0.001
ENSAT stage	—	—	0.900
I-II	128 (26.12%)	102 (26.42%)	—
III-IV	348 (71.02%)	271 (70.21%)	—
Unknown	14 (2.86%)	13 (3.37%)	—
Metastatic disease at diagnosis	—	—	0.891
No	298 (60.82%)	233 (60.36%)	
Yes	192 (39.18%)	153 (39.64%)	—
Surgery performed			<0.001
No	137 (27.96%)	152 (39.38%)	—
Yes	340 (69.39%)	229 (59.33%)	—
Unknown	13 (2.65%)	5 (1.30%)	—
Radiotherapy	—	—	0.058
No	388 (79.18%)	325 (84.20%)	—
Yes	102 (20.82%)	61 (15.80%)	—
Chemotherapy	—	—	<0.001
No	233 (47.55%)	246 (63.73%)	—
Yes	257 (52.45%)	140 (36.27%)	—
Receipt of regional lymph node surgery	—	—	0.008
No	374 (76.33%)	323 (83.68%)	—
Yes	101 (20.61%)	49 (12.69%)	—
Unknown	15 (3.06%)	14 (3.63%)	—
Marital status at diagnosis	—	—	0.247
Unmarried	209 (42.65%)	144 (37.31%)	—
Married	262 (53.47%)	223 (57.77%)	—
Unknown	19 (3.88%)	19 (4.92%)	—
Laterality	—	—	0.602
Left	260 (53.06%)	210 (54.40%)	—
Right	213 (43.47%)	167 (43.26%)	—
Unknown	17 (3.47%)	9 (2.33%)	—
Tumor size	—	—	0.417
≤5 cm	55 (11.22%)	54 (13.99%)	—
>5 cm	395 (80.61%)	298 (77.20%)	—
Unknown	40 (8.16%)	34 (8.81%)	—
Race	—	—	0.065
White	300 (61.22%)	218 (56.48%)	—
Hispanic	102 (20.82%)	73 (18.91%)	—
Asian or Pacific Islande	46 (9.39%)	58 (15.03%)	—
Black	42 (8.57%)	37 (9.59%)	—
Histologic grade	—	—	0.620
I-II	20 (4.08%)	15 (3.89%)	—
III-IV	53 (10.82%)	50 (12.95%)	—
Unknown	417 (85.10%)	321 (83.16%)	—

**FIGURE 2:**
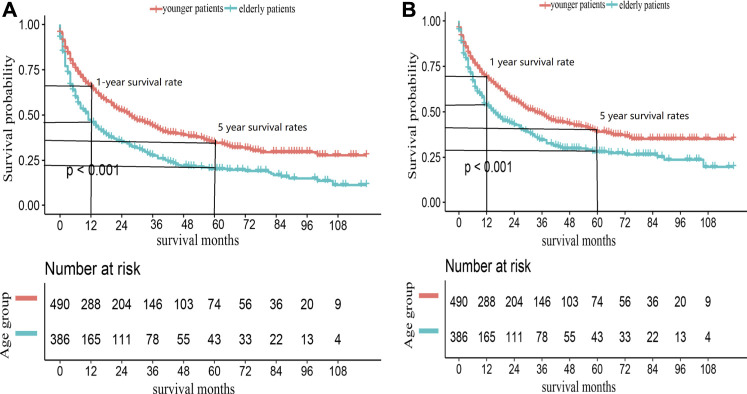
Kaplan-Meier survival curves for **(A)** OS and **(B)** CSS in ACC patients, according to age groups.

Elderly patients had inferior OS and CSS than younger ones (*p* < 0.001, Figure 2). The 1- and 5-year OS of elderly patients compared to younger patients were 46% [95% confidence interval (CI): 41%–52%] vs. 65% (95% CI: 62%–70%) and 20% (95% CI: 16%–25%) vs. 35% (95% CI: 30%–40%). The 1- and 5-year CSS were 54% (95% CI: 48%–59%) vs. 69% (95% CI: 65%–74%) and 28% (95% CI: 23%–34%) vs. 39% (95% CI: 34%–45%). The univariate analysis showed that metastatic disease, higher grade, and ENSAT stage were associated with decreased 1- and 5-year survival (Table 2).

**TABLE 2:** Univariate analysis of affecting overall and cancer specific survival of ACC patients.

Variables	OS	CSS
	HR (95% CI) *p*-value	HR (95% CI) *p*-value
Age		
<60	1.0	1.0
≥60	1.60 (1.35, 1.88) <0.001	1.46 (1.22, 1.75) <0.001
Sex		
Female	1.0	1.0
Male	1.16 (0.98, 1.37) 0.086	1.16 (0.97, 1.40) 0.11
Metastatic disease at diagnosis		
No	1.0	1.0
Yes	3.86 (3.25, 4.59) <0.001	4.53 (3.75, 5.48) <0.001
Surgery performed		
No	1.0	1.0
Yes	0.19 (0.16, 0.22) <0.001	0.17 (0.14, 0.21) <0.001
Unknown	0.27 (0.15, 0.48) <0.001	0.23 (0.12, 0.45) <0.001
Radiotherapy		
No	1.0	1.0
Yes	0.64 (0.50, 0.80) <0.001	0.69 (0.54, 0.88) 0.003
Chemotherapy		
No	1.0	1.0
Yes	1.03 (0.87, 1.22) 0.71	1.15 (0.96, 1.38) 0.12
Receipt of regional lymph node surgery		
No	1.0	1.0
Yes	0.69 (0.54, 0.87) 0.002	0.70 (0.54, 0.90) 0.006
Unknown	1.98 (1.33, 2.94) <0.001	1.71 (1.08, 2.71) 0.02
Marital status at diagnosis		
Unmarried	1.0	1.0
Married	0.95 (0.80, 1.12) 0.52	0.97 (0.81, 1.17) 0.79
Unknown	1.02 (0.67, 1.56) 0.92	0.97 (0.61, 1.56) 0.90
Laterality		
Left	1.0	1.0
Right	1.06 (0.90, 1.26) 0.49	1.04 (0.87, 1.26) 0.64
Unknown	3.14 (2.07, 4.76) <0.001	3.25 (2.08, 5.08) <0.001
Tumor size		
≤5 cm	1.0	1.0
>5 cm	1.03 (0.80, 1.33) 0.82	1.03 (0.78, 1.37) 0.81
Unknown	2.13 (1.51, 3.00) <0.001	2.15 (1.48, 3.13) <0.001
Race		
White	1.0	1.0
Hispanic	1.15 (0.94, 1.42) 0.18	1.09 (0.87, 1.37) 0.46
Asian or Pacific Islande	0.99 (0.76, 1.28) 0.92	0.96 (0.72, 1.28) 0.79
Black	0.91 (0.67, 1.23) 0.53	0.82 (0.58, 1.15) 0.24
ENSAT stage		
I-II	1.0	1.0
III-IV	3.21 (2.58, 3.99) <0.001	3.57 (2.80, 4.57) <0.001
Unknown	1.94 (1.14, 3.28) 0.01	1.55 (0.80, 3.00) 0.19
Histologic grade		
I-II	1.0	1.0
III-IV	1.98 (1.18, 3.33) 0.01	2.27 (1.27, 4.06) 0.006
Unknown	2.16 (1.35, 3.47) 0.001	2.29 (1.34, 3.90) 0.002

The multivariate analysis demonstrated that the factors independently linked to worsened OS were age ≥60 [HR: 1.47 (1.24–1.75)], metastatic disease [HR: 1.90 (1.48–2.43)], higher grade [HR: 1.71 (1.01–2.89)], and ENSAT stage [HR: 1.93 (1.49–2.51)]; and worsened CSS included age ≥60 [HR: 1.33 (1.24–1.75)], metastatic disease [HR: 2.12 (1.62–2.77)], higher grade [HR: 1.94 (1.08–3.46)], and ENSAT stage [HR: 1.99 (1.48–2.66)] (Table 3). Sex was not directly linked to survival.

**TABLE 3:** Multivariable cox regression of affecting overall and cancer specific survival of ACC patients.

Variables	OS	CSS
	HR (95% CI) *p*-value	HR (95% CI) *p*-value
Age ≥60	1.47 (1.24–1.75) <0.001	1.33 (1.24–1.75) <0.001
Sex	1.18 (1.00–1.40) 0.05	1.17 (0.97–1.41) 0.09
Metastatic disease at diagnosis	1.90 (1.48–2.43) <0.001	2.12 (1.62–2.77) <0.001
Surgery performed		
No	1.0	1.0
Yes	0.30 (0.24–0.38) <0.001	0.32 (0.26–0.41) <0.001
Unknown	0.41 (0.22–0.75) 0.004	0.41 (0.22–0.75) 0.01
Radiotherapy	0.73 (0.57–0.92) 0.009	0.76 (0.59–0.98) 0.04
Chemotherapy	0.72 (0.60–0.86) 0.001	0.76 (0.59–0.98) 0.01
Receipt of regional lymph node surgery		
No	1.0	—
Yes	1.12 (0.86–1.46) 0.39	—
Unknown	1.90 (1.22–2.93) 0.004	—
Tumor size		
≤5 cm	1.0	—
>5 cm	1.18 (0.91–1.54) 0.21	—
Unknown	0.90 (0.62–1.32) 0.60	—
ENSAT stage		
I-II	1.0	1.0
III-IV	1.93 (1.49–2.51) <0.001	1.99 (1.48–2.66) <0.001
Unknown	1.29 (0.72–2.32) 0.40	1.09 (0.56–2.12) 0.80
Histologic grade		
I-II	1.0	1.0
III-IV	1.71 (1.01–2.89) 0.04	1.94 (1.08–3.46) 0.03
Unknown	1.81 (1.12–2.91) 0.02	1.85 (1.08–3.18) 0.02

**FIGURE 3:**
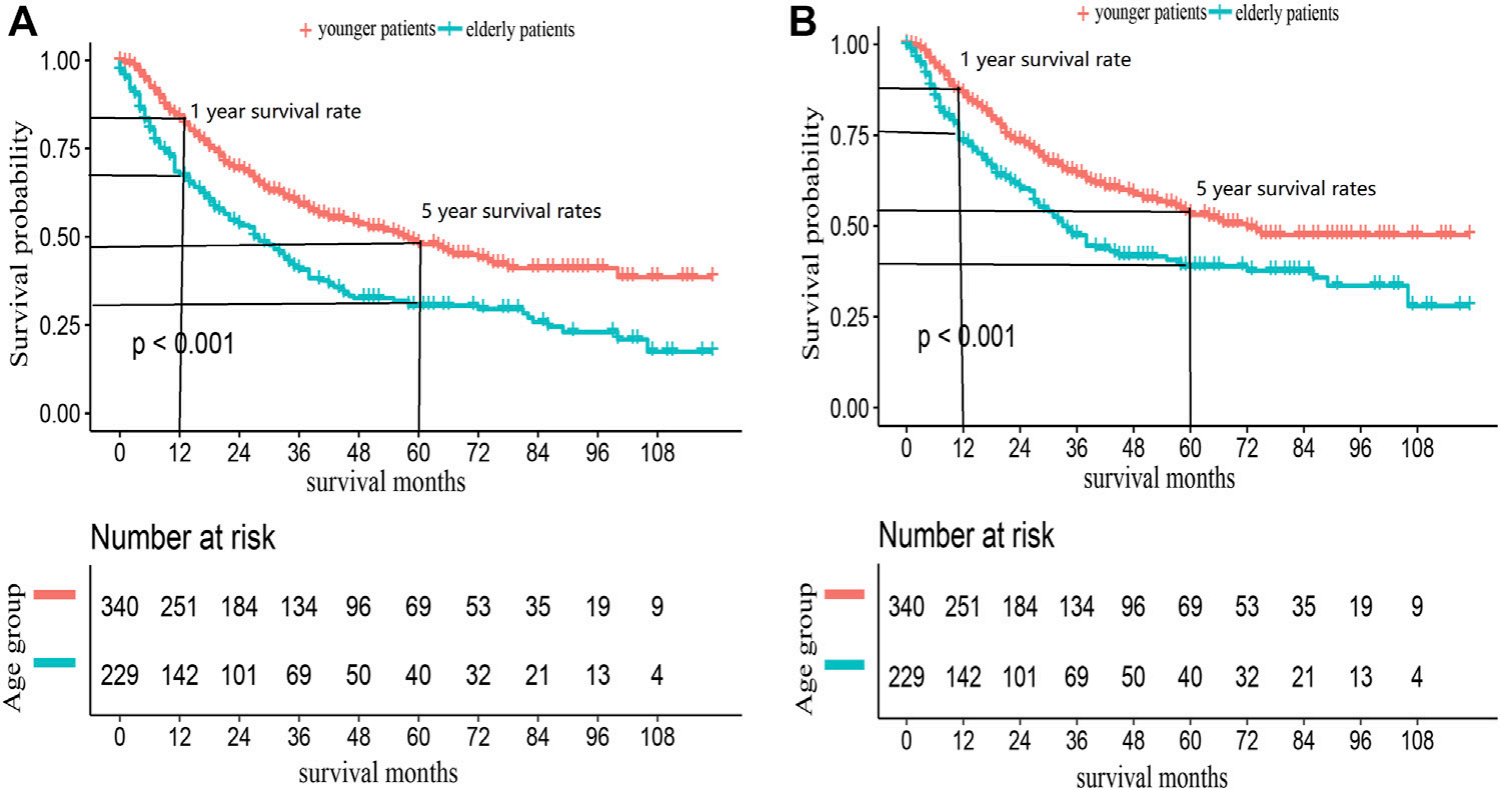
Kaplan-Meier survival curves for **(A)** OS and **(B)** CSS in ACC patients received surgery, according to age groups.

**FIGURE 4:**
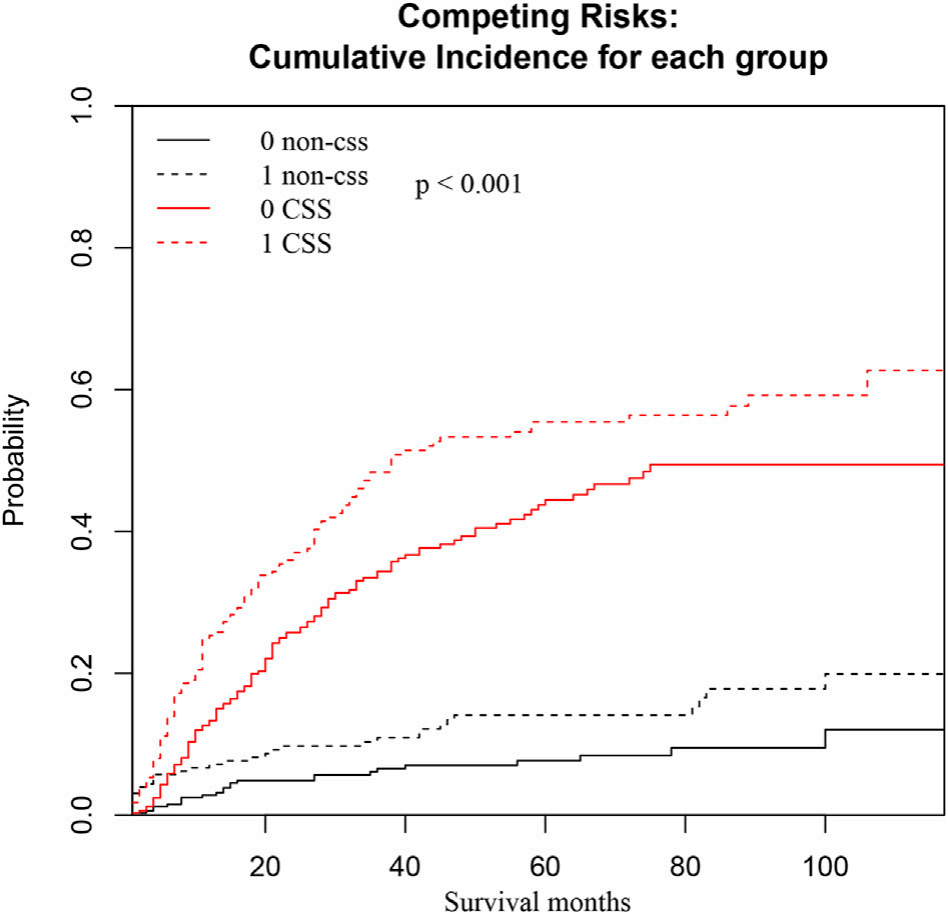
CSS and non-CSS cumulative incidence function curves based on the age groups. The black line represents non-cancer-specific death, the black dashed line represents elderly patients, the black solid line represents young patients, and the black dashed line above the black solid line represents the non-cancer-specific death rate of elderly patients with prolongation of survival time. The probability is higher than that of younger patients. The red line represents cancer-specific death, the red dashed line represents elderly patients, the red solid line represents younger patients, and the red dashed line above the red solid line represents that as the survival time increases, the probability of cancer-specific death in elderly patients is higher than that in younger patients.

We also conducted a subgroup analysis of patients who received surgery (Table 4). Patients with ≥60 years had an inferior OS and CSS than those under 60 (*p* < 0.001, Figure 3). The 1 and 5-year OS of elderly patients compared to younger patients were 68% (95% CI: 62%–74%) vs. 84% (95% CI: 80%–88%) and 30% (95% CI: 24%–38%) vs. 48% (95% CI: 42%–54%). The 1 and 5-year CSS were 73% (95% CI: 67%–80%) vs. 86% (95% CI: 83%–90%) and 40% (95% CI: 32%–47%) vs. 53% (95% CI: 47%–60%). The CIF curves displayed cumulative incidence rates for CSS beyond other causes, regardless of age group (Figure 4). Besides, the cumulative incidence rate of cancer-specific mortality of elderly patients was beyond younger patients.

**TABLE 4:** Subgroup analysis of OS and CSS in patients received surgery by multivariable cox regression.

Variables	OS	CSS
	HR (95% CI) *p*-value	HR (95% CI) *p*-value
Age ≥60	1.94 (1.53–2.46) <0.001	1.80 (1.39–2.33) <0.001
Sex	1.31 (1.03–1.66) 0.03	1.33 (1.02–1.73) 0.03
Metastatic disease at diagnosis	1.82 (1.35–2.45) <0.001	2.07 (1.51–2.84) <0.001
Radiotherapy	0.63 (0.46–0.85) 0.003	0.69 (0.50–0.96) 0.03
Receipt of regional lymph node surgery		
No	1.0	—
Yes	1.10 (0.84–1.44) 0.50	—
ENSAT stage		
I-II	1.0	1.0
III-IV	2.24 (1.67–3.00) <0.001	2.33 (1.69–3.20) <0.001
Histologic grade		
I-II	1.0	1.0
III-IV	1.95 (1.08–3.50) 0.03	2.30 (1.18–4.50) 0.01

## Discussion

In the elderly, ACC is uncommon, aggressive, and has a dismal prognosis. Little research has examined the prognosis of ACC in older patients. Li et al. (2018) recently reported that the survival rates of elderly patients (60–89 years old) were lower than younger patients. Herein, we compared elderly and younger patients to identify differences between these two cohorts and explored the factors independently related to survival.

Comparing younger patients to elderly patients, we found that the former had much higher survival rates. We also found that baseline tumor features between elderly and younger adults did not significantly differ, except in the proportion of patients who underwent surgery, regional lymph node surgery, and chemotherapy. However, older patients had markedly worse OS and CSS than younger ones, as demonstrated by multivariate analysis, where patients with ≥60 years had a higher risk of death. The only treatment for ACC, regarding its development, is total tumor removal. Nevertheless, elderly patients are less likely to undergo surgery and lymph node dissection than younger patients (Huisman et al., 2017). Previous research has offered a few potential explanations for this outcome. The choice of surgery for older patients is influenced by various circumstances, including concomitant illness, diminished functional status, mental status changes, resource constraints, a sense of having a limited life expectancy, and an imagined incapacity to tolerate treatment (Kemeny et al., 2000). For race, most ACC patients were white, regardless of age, consistent with previous research (Tella et al., 2018; Holoubek et al., 2022). We also found that men were more likely to develop the disease than women, independent of age, which has also been reported in other studies (Luton et al., 1990; Scollo et al., 2016; Li et al., 2018). The ACC fraction was larger on the left side than the right, and prior investigations have also found this odd laterality discrepancy (Luo et al., 2017; Wang et al., 2017; Sharma et al., 2018). However, the precise mechanisms underlying this phenomenon remain unknown (Fassnacht et al., 2011). Additionally, most ACCs had high tumor grades and advanced ENSAT stages. One explanation is that due to the comparatively low specificity of current diagnostic instruments, ACCs at lower tumor grades and earlier ENSAT stages might be mistakenly categorized as benign knubs. ACC is only found once tumors have advanced to a higher grade and stage (Conzo et al., 2009).

Although the proportion of older patients who underwent surgery was lower than that of younger patients, the multivariate analysis showed that complete tumor resection was strongly related to the OS and CSS. This result demonstrated that surgical excision of the original tumor continues to be the primary treatment for older ACC patients. According to previous research, complete tumor excision is crucial for survival and preventing recurrence, consistent with our results (Sinclair et al., 2020). The justification might be that, since ACC is extremely malignant and advances quickly, surgical excision is frequently the only option for treating local illnesses. Additionally, surgical intervention might increase the survival of advanced patients. The literature on the role of lymphadenectomy and ACC is inconsistent and heterogeneous. Our multivariate analysis showed that locoregional lymphadenectomy (LND) did not lead to survival advantage, similar to previous research (Alanee et al., 2015; Saade et al., 2015; Sada et al., 2021). However, according to several studies, LND in stage I to III ACC has an oncological advantage (Reibetanz et al., 2012; Saade et al., 2015; Gerry et al., 2016). Lymph node involvement is a bad predictor of tumor recurrence and disease-free and disease-specific survival. Therefore, removing the involved lymph nodes as part of a thorough lymphadenectomy during primary surgery is reasonable. Nevertheless, the LND rate in elderly patients was only 12% (Table 1), similar to (Deschner et al., 2020). Older patients can have more co-morbidities, making it difficult to undergo extensive surgery that includes LND. Prospective studies are required to understand better the potential value of preventive nodal dissection for ACC in older patients.

There is also debate on the roles of radiotherapy and chemotherapy in ACC. Although radiotherapy and chemotherapy have some effects on the locoregional management of ACC in adults, how they affect senile illnesses remains unclear (Polat et al., 2009; Al Asadi et al., 2021). According to Wajchenberg BL, radiation is typically considered insensitive to ACC, increasing the risk of subsequent cancers (Wajchenberg et al., 2000). However, our multivariate analysis showed that radiotherapy is significantly related to the OS and CSS, even in subgroup analysis, consistent with other studies (Sabolch et al., 2011; Habra et al., 2013; Wu et al., 2021). A recent meta-analysis gathered information from earlier retrospective studies and focused on adjuvant radiotherapy following surgical resection for ACC (Viani and Viana, 2019). Adjuvant radiotherapy can be provided safely, with few side effects, and reduce local recurrence, but it has no meaningful impact on distant metastases, according to a previous meta-analysis. Besides, the frequency of radiotherapy-related side effects should be carefully followed, particularly in elderly patients. Jiawei Zhu systematically reviewed the literature about adjuvant radiotherapy for ACC patients and showed that the adverse effects of radiotherapy were considered mild, leading to a high therapeutic index (Zhu et al., 2020). In the future, a large-scale randomized controlled trial (RCT) is required to prove these advantages of radiotherapy.

Moreover, we found that chemotherapy was associated with better OS and CSS, similar to the retrospective multicenter cohort study by Otilia Kimpel, indicating that adjuvant chemotherapy might be related to prolonged recurrence-free and overall survival in ACC patients (Kimpel et al., 2021). Martin Fassnach found that the OS rates in ACC patients who received combination treatment of etoposide, doxorubicin, cisplatin, and mitotane remained dismal (Fassnacht et al., 2012). However, the combination treatment could significantly increase the progression-free survival of patients. Future prospective, randomized trials, such as ADIUVO-2, will determine the precise function of adjuvant chemotherapy in ACC patients.

In our current research, 39.64% of elderly patients had metastatic disease at the time of diagnosis. Also, our survival analysis showed that metastatic disease was related to lower survival. This finding is consistent with earlier research that showed metastatic disease and a more advanced stage to be independent predictive factors for OS and CSS (Ayala-Ramirez et al., 2013). Zachary Klaassen showed that single, divorced, or widowed (SDW) patients have much higher cancer-specific death rates than married patients (Klaassen et al., 2015). However, in our study, marital status was not related to survival in ACC patients. ACC patients of various ages were included in the Zachary Klaassen study (adults and pediatrics), and pediatric patients might overstate the effect of SDW on patient survival. Thus, more research needs to assess marital status’s role in the survival of ACC patients.

However, our current study also has some limitations. First, this was an observational study. Thus, we could not avoid the weakness of retrospective research. Second, since the data came from several hospitals, it is impossible to do a pathologic review of every specimen. Pathologic variables influence how well each hospital’s analysis is done. Third, SEER did not include information on patient comorbidities, performance status, kind of targeted treatment, the timing of surgery, or relevant molecular markers that might have been useful prognostic indicators for ACC patients.

## Conclusion

Herein, we used a huge national database to assess the prognosis of elderly ACC patients and compare surgical outcomes and survival between elderly and younger patients. We found that older patients had lower OS and CSS at 1 and 5 years compared to younger patients, although there was no obvious difference in baseline tumor information. Age ≥60, metastatic disease, higher grade, and ENSAT stage were independently related to survival. Furthermore, future prospective studies are required to establish the specific role of adjuvant radiation and chemotherapy in elderly ACC patients.

## Data Availability

The raw data supporting the conclusion of this article will be made available by the authors, without undue reservation.
